# Rickettsial Infections and Fever, Vientiane, Laos

**DOI:** 10.3201/eid1202.050900

**Published:** 2006-02

**Authors:** Simaly Phongmany, Jean-Marc Rolain, Rattanaphone Phetsouvanh, Stuart D. Blacksell, Vimone Soukkhaseum, Bouachanh Rasachack, Khamphong Phiasakha, Surn Soukkhaseum, Khamthavi Frichithavong, Vang Chu, Valy Keolouangkhot, Bertrand Martinez-Aussel, Ko Chang, Chirapha Darasavath, Oudayvone Rattanavong, Siho Sisouphone, Mayfong Mayxay, Sisouphane Vidamaly, Philippe Parola, Chanpheng Thammavong, Mayboun Heuangvongsy, Bounkong Syhavong, Didier Raoult, Nicholas J. White, Paul N. Newton

**Affiliations:** * Mahosot Hospital, Vientiane, Laos;; † Université de la Méditerranée, Marseille, France;; ‡ University of Oxford, Oxford, United Kingdom;; § Mahidol University, Bangkok, Thailand;; # National University of Laos, Vientiane, Laos;; ¶ Francophone Institute for Tropical Medicine, Vientiane, Laos

**Keywords:** Laos, typhus, fever, Orientia tsutsugamushi, Rickettsia typhi, Rickettsia helvetica, Rickettsia felis, Rickettsia conorii, Rickettsia AT1

## Abstract

Rickettsial diseases have not been described previously from Laos, but in a prospective study, acute rickettsial infection was identified as the cause of fever in 115 (27%) of 427 adults with negative blood cultures admitted to Mahosot Hospital in Vientiane, Laos. The organisms identified by serologic analysis were *Orientia tsutsugamushi* (14.8%), *Rickettsia typhi* (9.6%), and spotted fever group rickettsia (2.6% [8 *R. helvetica*, 1 *R. felis*, 1 *R. conorii* subsp. *indica*, and 1 *Rickettsia* "AT1"]). Patients with murine typhus had a lower frequency of peripheral lymphadenopathy than those with scrub typhus (3% vs. 46%, p<0.001). Rickettsioses are an underrecognized cause of undifferentiated febrile illnesses among adults in Laos. This finding has implications for the local empiric treatment of fever.

## Abstract 2

*Rickettsia* spp. are an underrecognized cause of undifferentiated febrile illness.

The Lao People's Democratic Republic (Laos) is situated mostly east of the Mekong River and borders Thailand, Cambodia, Burma (Myanmar), China, and Vietnam. Most (83%) of the population of 5.2 million are rural rice farmers, the per capita income is US $326/year, and life expectancy is 54 years (*1*). Although more data have been obtained in wealthier countries in Asia, minimal information exists on the clinical epidemiology of infectious disease in Laos.

The etiology of fever in Laos usually remains obscure because of limited laboratory diagnostic facilities. In 2000, the main differential diagnoses for adults admitted with fever to the hospital in Vientiane, the capital, were slide-positive malaria or slide-negative *syndrôme paludéen*, or malaria syndrome: both were treated with antimalarial drugs and the latter with additional antimicrobial drugs (unpub. data). Rickettsial diseases, caused by *Orientia tsutsugamushi* (scrub typhus), *Rickettsia typhi* (murine typhus), and members of the spotted fever group (SFG), cause fever in Thailand, Malaysia, China, and Vietnam (*2**–**5*), and their public health consequences have recently been emphasized in Sri Lanka (*6*) and Nepal (*7*). Indonesian peacekeeping troops seroconverted to *O. tsutsugamushi* and *R. typhi* during their residence in Cambodia (*8*), but acute, symptomatic infections with rickettsia have not been described there since the 1930s (*9*).

No studies that examined the causes of fever in Laos, which has economic, cultural, and geographic differences from adjoining countries, have been published. Such information is crucial in developing appropriate diagnostic tests and guidelines, determining empiric treatment for nonmalarious fever, and planning public health interventions. The mite vectors of scrub typhus have been described from Laos (*10*), but no rickettsial disease has been described from the country, apart from the seroconversion of US troops to *O. tsutsugamushi* (*11*). Therefore, we conducted a 2-year prospective study of the causes of fever among adults admitted to Mahosot Hospital, who were both blood-culture and malaria-smear negative, to determine the causes of *syndrôme paludéen*. We describe the serologic test results for rickettsiae.

## Methods

### Study Site and Patients

The study was conducted at Mahosot Hospital, Vientiane, a 365-bed primary- to tertiary-care hospital that specializes in internal medicine, which has ≈1,200 admissions per month. This hospital, along with 4 other major hospitals (1,210 beds total) and local provincial and district hospitals, serves a population of ≈900,000 people, including the urban population of Vientiane City and surrounding farming communities of Vientiane Province, and less frequently, outlying provinces. We recruited patients admitted from November 2001 to October 2003 on all 4 adult medical wards (including an adult intensive care unit), making up 91 beds. Ethical clearance was granted by the Faculty of Medical Sciences Ethical Review Committee, National University of Laos.

### Clinical Procedures

All adults (>15 years of age) admitted with fever had blood cultures taken if community-acquired septicemia was suspected and they gave verbal informed consent. If the patient came from an area of Laos with endemic malaria, Giemsa-stained malaria thick and thin films were examined. If the blood culture showed no clinically meaningful growth after 3 days of incubation, the malaria film was negative, and the patient gave verbal informed consent, a 5-mL whole blood sample was taken for serum analysis. An additional 5-mL convalescent-phase venous blood sample was collected ≈1 week later. The presence of eschars was not recorded systematically, since without evidence of rickettsial infection they were not routinely looked for. Patients' conditions were further investigated and treated according to local hospital practice.

### Laboratory Procedures

Serum samples were stored at –80°C until analysis. Specific microimunofluorescence (IFA) assays were performed in Marseille, France, by using whole-cell antigens of *O. tsutsugamushi* serotypes Karp, Kato, Gilliam, and Kawasaki (*12*) and with *Bartonella henselae*, *Coxiella burnetii*, *R. conorii* subsp. *indica*, *R. felis*, *R. heilongjiangensis*, *R. helvetica*, *R. honei*, *R. japonica*, *Rickettsia* "ATI," *R. slovaca*, and *R. typhi* (*13*–*15*). An IFA result was considered positive if any of the following were detected: 1) positive antibody titers >1:128 for immunoglobulin G (IgG) and >1:64 for IgM, 2) seroconversion, or 3) >4-fold increase in titers between acute- and the convalescent-phase serum (*5**,**13*). Western immunoblotting was performed on samples positive for *Rickettsia* spp. both before and after cross-absorption with relevant antigens (*12**,**13*). Full blood counts (n = 364) and serum biochemical test results (n = 352) were analyzed on Abx MICROSOT (Abx Hematologie, Montpellier, France) and Cobas Integra (Roche Co. & Tegimenta Ltd, Rotkreuz, Switzerland) analyzers, respectively.

### Statistical Analysis

Analysis was performed by using Stata v. 8 (StataCorp LP, College Station, TX, USA). Categoric variables were compared with Fisher exact test and continuous variables by Student *t* test and Mann-Whitney U test as appropriate. Multivariate logistic regression (backwards) was performed to evaluate variables associated with serologic diagnoses.

## Results

### Serology

During the 2 years of the study, 466 adults were recruited; clinical and laboratory data, including rickettsial serology, were available for 427. Forty-five patients (12.6%) did not have a prior blood culture, and 218 (51%) had a convalescent-phase serum sample taken (median 5 [range 1–50] days after the admission sample).

Of 427 patients, serologic evidence for acute rickettsial infections were found in 115 (26.9%): *O. tsutsugamushi* in 63 (14.8%), *R. typhi* in 41 (9.6%), and SFG rickettsiae in 11 (2.6% [8 *R. helvetica*, 1 *Rickettsia* "AT1," 1 *R. felis*, and 1 *R. conorii* subsp. *indica*]). No serologic evidence was found for acute *B. henselae*, *C. burnetii*, *R. heilongjiangensis*, *R. honei*, *R. japonica*, or *R. slovaca* infection. Of the 63 patients with serologic evidence of infection with *O. tsutsugamushi*, the highest titers were with the Gilliam serotype for 9 patients, the Gilliam or Kawasaki serotype in 9, the Gilliam or Kato serotype in 6, and all 3 serotypes in 39.

### Clinical Features

Patients with scrub typhus could not be distinguished reliably from those with murine typhus at the bedside or in retrospective review of all clinical and laboratory details (Table 1). Patients with scrub typhus had a higher frequency of lymphadenopathy and abnormal chest examination than patients with murine typhus (p<0.001 and p = 0.002, respectively). The respiratory rate was faster for those with scrub typhus than those with murine typhus (p = 0.0012). Multiple logistic regression suggested that, in comparison to patients with murine typhus, the presence of lymphadenopathy and a faster respiratory rate were independently associated with scrub typhus (lymphadenopathy, abnormal chest examination, and respiratory rate were entered). Raised (>90 IU/L) serum creatinine kinase concentrations were found in 273 (63.9%) of patients in the serologic study: 57% with scrub typhus, 63% with murine typhus, and 50% with positive *R. helvetica* serologic test results. Patients with rickettsioses who had myalgia on admission had significantly higher serum creatinine kinase (geometric mean 119 IU/L, 95% confidence interval [CI] 90–158) concentrations than those who did not (geometric mean 47 IU/L, 95% CI 26–87) (p = 0.02).

**Table 1:** Admission clinical features of 104 Lao adults with serologic evidence of acute murine and scrub typhus*

Variable	Murine typhus (n = 41)†	Scrub typhus (n = 63)†	p value	Reference range
Age, (y)‡	40 (17–70)	31 (16–73)	0.5	
No. (%) male	26 (63)	40 (63)	0.6	
No. days ill‡	11 (3–30)	10 (2–42)	0.2	
Headache (%)	38 (95) (n = 40)	60 (95)	1.0	
Abdominal pain (%)	17 (43) (n = 40)	22 (35)	0.5	
Nausea (%)	18 (45) (n = 40)	39 (62)	0.09	
Vomiting (%)	11 (28) (n = 40)	25 (40)	0.3	
Diarrhea (%)	7 (18) (n = 40)	22 (35)	0.07	
Cough (%)	14 (35) (n = 40)	24 (38)	0.8	
Sputum (%)	8 (20) (n = 40)	13 (21)	1.0	
Dyspnea (%)	5 (13) (n = 40)	7 (11)	1.0	
Chest pain (%)	3 (8) (n = 40)	13 (21)	0.1	
Back pain (%)	15 (38) (n = 40)	19 (30)	0.5	
Dysuria (%)	3 (8) (n = 40)	2 (3)	0.4	
Arthralgia (%)	10 (25) (n = 40)	13 (21) (n = 62)	0.6	
Myalgia (%)	34 (85) (n = 40)	59 (95) (n = 62)	0.1	
Sore throat (%)	3 (8) (n = 40)	12 (19)	0.2	
Lymphadenopathy (%)	1 (3) (n = 38)	27 (46) (n = 59)	<0.001	
Bleeding (%)	2 (5) (n = 39)	4 (6) (n = 58)	1.0	
Convulsions (%)	0 (n = 40)	1 (2) (n = 62)	1.0	
Rash (%)	5 (13) (n = 38)	16 (27) (n = 59)	0.1	
Abnormal chest exam (%)	1 (3) (n = 38)	16 (27) (n = 59)	0.002	
Abdominal tenderness (%)	1 (3) (n = 39)	6 (10) (n = 59)	0.2	
Liver palpable (%)	27 (73) (n = 37)	30 (52) (n = 58)	0.05	
Spleen palpable (%)	6 (15) (n = 39)	9 (15) (n = 59)	1.0	
Temperature (°C)‡	38.5 (38.2–38.8) (n = 36)	38.6 (38.4–38.9) (n = 56)	0.3	
Pulse/min§	91.1 (87.1–95.1) (n = 36)	95.7 (91.8–99.6) (n = 54)	0.09	
Systolic blood pressure (mm Hg)‡	100 (80–130) (n = 36)	100 (90–150) (n = 54)	0.6	
Diastolic blood pressure (mm Hg)‡	70 (50–80) (n = 36)	65 (50–110) (n = 54)	0.8	
Respiratory rate/min§	20.7 (19.9–21.6) (n = 36)	22.9 (22.0–23.8) (n = 58)	0.0012	
Glasgow Coma Score‡	15 (15) (n = 38)	15 (7–15) (n = 59)	0.3	
Meningism (%)	2 (5) (n = 39)	7 (12) (n = 59)	0.3	
Hematocrit (%)‡	40 (13–48) (n = 35)	40 (23–50) (n = 53)	0.7	
Leukocyte count (×10^9^/L)‡	10.4 (3.1–38) (n = 37)	11.8 (0.7–26.3) (n = 54)	0.1	4.0–11.0
Neutrophils (%)‡	68 (26-86) (n = 37)	70 (0–93) (n = 54)	0.2	
Platelets (×10^9^/L)§	190 (23–350) (n = 37)	200 (192–208) (n = 49)	0.4	150–400
Serum creatinine (μmol/L)‡	106 (70–466) (n = 32)	106 (70–783) (n = 53)	0.7	53–123
Serum AST (IU/L)‡	87 (32–789) (n = 31)	86 (16–437) (n = 52)	0.6	7–35
Serum ALT (IU/L)‡	39 (20–234) (n = 31)	48 (12–180) (n = 52)	0.7	7–35
Serum albumin (g/L)‡	38 (26–50) (n = 32)	35 (22–49) (n = 53)	0.2	35–50
Serum creatinine kinase (IU/L)¶	113 (80–159) (n = 32)	121 (70–210) (n = 53)	0.8	24–190
Serum alkaline phosphatase (IU/L)‡	156 (47–532) (n = 32)	175 (55–745) (n = 53)	0.5	120–290
Serum direct bilirubin (μmol/L)‡	8.3 (1.7–60.4) (n = 32)	8.3 (2.6–83.0) (n = 52)	0.9	0.5–8.8
Serum total bilirubin (μmol/L)‡	17.9 (7.7–109) (n = 32)	18.4 (7.7–131) (n = 52)	0.4	1.7–20
No. patients serum total bilirubin >50 μmol/L (%)	2 (6) (n = 32)	4 (8) (n = 52)	1.0	
No. patients serum AST >105 IU/L (%)	11 (36) (n = 31)	18 (35) (n = 52)	1.0	
No. patients serum ALT >105 IU/L (%)	6 (19) (n = 31)	5 (10) (n = 52)	0.3	
Deaths (%)	0	1 (1.5)		

*AST, aspartate aminotransferase; ALT, alanine aminotransferase; CI, confidence interval. †The available sample size is given in parentheses where the entire sample was not available for a given variable. ‡Median (range). §Mean (95% CI). ¶Geometric mean (95% CI).

Seventeen patients with scrub typhus (27.0%) had evidence for severe organ dysfunction; 7 (11.9%) of 59 had meningismus, 7 (11.1%) of 63 had dyspnea, and 7 (13.2%) of 53 had a serum creatinine level >133 μmol/L. While 4 (7.7%) of 52 patients had a total serum bilirubin level >50 μmol/L, 18 (34.6%) of 52 had a serum aspartate aminotransferase (AST) level >3 times the upper limit of the reference range. Nine patients with murine typhus and severe organ dysfunction (22.5% of 40 patients with data) were also encountered; 2 (5.1%) of 39 had meningism, 5 (12.5%) of 40 had dyspnea, and 2 (6.3%) of 32 had a serum creatinine level >133 μmol/L.

Of the 8 patients with serologic evidence of acute *R. helvetica* infection, 6 had headache, 4 had vomiting, 1 had diarrhea, 2 had cough, 2 had dyspnea, 7 had myalgia, 4 had a palpable liver, and none had palpable lymphadenopathy or splenomegaly (Tables 2 and 3). One had a petechial rash at admission, and rash developed in 1 patient 2 days after admission. The median (range) serum biochemistry results for patients with *R. helvetica* infection were creatinine 85 (67–142) μmol/L, AST 84 (35–118) IU/L, alanine aminotransferase (ALT) 50 (14–87) IU/L, albumin 39 (23–45) g/L, creatinine kinase 49 (16–125) IU/L, alkaline phosphatase 115 (96–217) IU/L, direct bilirubin 4.8 (3.7–7.3) μmol/L, and total bilirubin 9.5 (8.8–16.8) μmol/L. None of the 8 patients had a bilirubin level >50 μmol/L or an ALT level >3 times the upper limit of the reference range, but 2 patients had an AST level >3 times the upper limit of the reference range.

**Table 2:** Clinical features of patients with serologic evidence for acute spotted fever rickettsioses admitted to Mahosot Hospital*

Patient no.	Age (y), sex	Occupation	Month of onset of illness	Clinical features	Home
45	30, male	Construction worker	March	18-day fever, myalgia, nausea, epistaxis, vomiting, abdominal pain, petechial rash on trunk and legs; liver and spleen not palpable; treated with ampicillin and gentamicin	Vientiane City
72	35, female	Teacher	April	13-day fever, chills, headache, nausea, myalgia, vomiting, conjunctival suffusion, dyspnea, 12-cm liver; treated with ofloxacin	Vientiane City
86	25, male	Health worker	May	11-day fever, headache, nausea, vomiting, abdominal pain, 10-cm liver	Vientiane City
114	18, male	Student	June	14-day fever, chill, headache, arthralgia, myalgia, rash developed 2 days after admission, 12-cm liver; treated with ofloxacin	Vientiane Province
198	50, male	Government official	September	24-day fever, headache, arthralgia, myalgia, vertigo, epistaxis, diarrhea; abdominal CT suggested hepatic carcinoma; no antimicrobial drug	Xieng Khuang Province
237	64, male	Government official	September	21-day fever, myalgia, arthralgia, abdominal pain, sore throat, cough, dyspnea; chest exam abnormal	Vientiane City
290	24, female	Construction worker	March	7-day fever, headache, vomiting, myalgia, unproductive cough, diarrhea; treated with doxycycline	Vientiane City
362	23, female	Student	June	10-day fever, myalgia, headache, conjunctival suffusion, 8-cm liver; treated with doxycycline	Vientiane City
297	43, female	Housewife	March	14-day fever, headache, jaundice, RUQ pain, myalgia, 8-cm hepatomegaly; abdominal CT suggested tumor of intrahepatic bile ducts (cholangiocarcinoma?); treated with ampicillin and gentamicin	Xieng Khuang Province
55	34, female	Housewife	April	7-day fever, chills, headache, myalgia, diarrhea, abdominal pain, nausea, vomiting, rash on arms and abdomen; treated with oral chloramphenicol	Vientiane Province
239	46, male	Merchant	November	6-day fever, headache, myalgia, arthralgia, nausea, abdominal pain, diarrhea, dyspnea, dry cough, and sore throat; treated with doxycycline	Vientiane City

*CT, computed tomographic scan; RUQ, right upper quadrant.

**Table 3:** Serologic results of patients with serologic evidence for acute spotted fever rickettsioses admitted to Mahosot Hospital

Patient no.	Immunofluorescence results (IgG/IgM admission sample, IgG/IgM convalescent-phase sample)*							
	*Rickettsia japonica*	*R. helvetica*	*R. heilongjiangensis*	*R. slovaca*	*R. felis*	*R. honei*	*R. conorii†*	"AT1"‡
45	0/0, 1:256/1:128	**0/0,** **1:1,024/1:256**	0/0, 1:1,024/1:128	0/0, 1:1,024/1:128	0/0, 1:256/1:256	0/0, 1:256/1:256	0/0, 0/1:256	0/0, 1:256/1:256
72	1:64/1:32	**1:128/1:32**	1:64/1:32	1:128/1:32	0/0	0/0	0/0	0/0
86	0/0, 1:64/0	**0/0,** **1:64/1:128**	0/0, 1:64/0	0/0, 1:64/1:128	0/1:128, 0/1:128	0/0, 0/1:32	0/0, 0/1:32	1:128/0, 1:128/1:32
114	1:128/1:64	**1:256/1:512**	1:128/1:64	1:256/1:512	0/1:32	0/0	0/0	0/0
198	0/0	**1:128/1:64**	0/0	0/1:32	0/0	1:128/0	1:64/0	1:256/0
237	1:128/0	**1:256/1:32**	1:128/0	1:256/1:32	0/0	0/1:32	0/1:32	0/1:64
290	0/1:32, 0/1:32	**1:64/1:32,** **1:64/1:32**	0/0, 0/0	0/0, 0/0	0/0, 0/0	0/0, 0/0	0/0, 0/0	0/1:32, 0/1:32
362	0/0, 0/0	**1:16/1:16,** **1:32/1:32**	0/0, 0/0	0/0, 0/0	1:16/1:16, 1:16/1:32	0/0, 0/0	0/0, 0/0	0/0, 1:32/1:32
297	0/0, 0/1:64	0/0, 0/1:64	0/0, 0/1:64	0/0, 0/1:64	0/0, 0/0	0/0, 0/1:64	0/0, 0/0	**0/0,** **0/1:64**
55	0/0, 1:64/1:32	0/1:64, 1:64/1:128	0/0, 1:64/1:32	0/1:64, 1:64/1:128	**0/0,** **1:256/1:128**	0/0, 1:256/0	0/0, 1:64/0	0/0, 1:256/0
239	0/0	0/0	0/0	0/0	1:64/0	1:64/1:32	**1:64/1:32**	1:64/1:32

*Titers in **boldface** indicate the pathogen considered to be responsible for the serologic response. †*R. conorii* subsp. *indica*. ‡*Rickettsia* "AT1" from Japan.

### Geographic Distribution

Districts in which patients lived were recorded for 417 (98%) patients in the serologic study; 73% lived in Vientiane City, and 22% Vientiane Province. The proportion of patients with a home address in Vientiane City was 71% for scrub typhus and 55% for murine typhus patients. Outside Vientiane City and Province, patients with scrub typhus came from Houaphanh and Borikhamxay Provinces, and patients with murine typhus came from Borikhamxay and Luang Prabang Provinces. Of the 11 patients with serologic evidence of spotted fever rickettsiosis, 7 were from Vientiane City, 2 from Vientiane Province, and 2 from Xieng Khuang Province.

### Outcome

Of 63 patients with scrub typhus for whom outcome is known, 1 (1.6%) died in the hospital. This 23-year-old housewife died 14 days after delivering a healthy girl at home; she had gone to the hospital with a 1-week history of fever before parturition. Pneumonia, vaginal bleeding from retained placenta, and hypotension developed; her Glasgow Coma Score was 7 of 15. In the hospital, she underwent uterine curettage and received ampicillin, gentamicin, azithromycin, ceftriaxone, and metronidazole. Fever developed in the daughter, and she died 4 days after her mother. The death rate among adults with serologic evidence of an acute rickettsiosis was therefore 1 in 115 (0.9%).

## Discussion

These serologic data suggest that scrub typhus and murine typhus are underrecognized causes of fever among adults in Vientiane. A wide diversity of rickettsiae were identified for the first time in Laos. Scrub typhus was the most common rickettsiosis identified. The patients tended to be young adult males presenting with fever, headache, nausea, myalgia, lymphadenopathy, and a palpable liver. Seventeen (27%) patients with scrub typhus had severe disease, and 18 (34.6%) had a liver biochemistry profile consistent with that of hepatitis. In a recent series of 462 patients with scrub typhus from Japan, lymphadenopathy, headache, myalgia, hepatomegaly, and eschar were recorded in 52%, 46%, 16%, 3%, and 87% of patients, respectively. Elevated serum AST and ALT levels were also common (87% and 77%, respectively) among these Japanese patients (*16*). In comparison to Lao patients, Japanese patients had a substantially lower prevalence of myalgia and hepatomegaly. The clinical importance of acute scrub typhus in the death of the Lao patient who also had retained placenta and probable intrauterine infection remains uncertain. Her infant may have died of neonatal scrub typhus (*17*). Of 12 case reports of scrub typhus in pregnancy (*17**–**19*), 8 recorded stillbirth, miscarriage, neonatal scrub typhus, or neonatal death, but all the mothers survived. During the 2 years of this study, patients with scrub typhus became ill in the late hot weather and monsoon, similar to observations made 60 years ago in Burma (*20*), but different from the geographically variable epidemiologic features noted in Japan (*16*). Recent clinical observations suggest that the prevalence of eschars in Lao patients with serologically confirmed scrub typhus when the entire skin surface is examined is ≈52% (unpub. data) and 0% in patients with confirmed murine typhus. Therefore, a thorough search for eschars will help with the diagnosis of scrub typhus.

Patients with murine typhus also tended to be young adult males with a clinical profile similar to those with scrub typhus but with a strikingly lower frequency of lymphadenopathy (3% vs. 46%). Similar proportions of patients with murine typhus and scrub typhus had raised serum bilirubin and AST levels. In a series of 137 patients with murine typhus in southern Thailand (*21*), 20% had skin rash, 24% had hepatomegaly, and 5% had splenomegaly. In contrast, among 83 Cretans, 80% had a rash, perhaps because it was easier to detect on fairer skin (*22*). A relatively low frequency of lymphadenopathy in patients with murine typhus has been described from Crete (4% [22]), Texas (16% of children [23]), and Spain (2% [24]). In the Lao series, cough was present in 35% of patients with murine typhus. Respiratory symptoms and signs have been reported among murine typhus patients with cough present in 59% (*25*), 15% (children [23]), 28% (*21*), and 25% (*24*) of patients. No concurrent comparisons have been made of clinical features of scrub and murine typhus at 1 site, but the Lao data suggest that the presence of peripheral lymphadenopathy, chest signs, and eschars are clinically useful signs that suggest scrub, rather than murine, typhus.

We also found serologic evidence for 4 SFG species. Although Western blotting and cross-absorbance studies were performed, evidence for rickettsiae in Laos is based on serologic methods and therefore, especially for SFG, needs to be confirmed by genetic analysis (*4*). Human SFG *Rickettsia* infections have been described in Thailand, China, Korea, Malaysia, and Japan (*3**,**4**,**26**,**27*) but not in Laos, Vietnam, Burma or Cambodia. Evidence for human *R. helvetica* infections has been found in Europe (*14**,**28**,**29*), Thailand (*5*), and possibly Australia or Japan (*30*). One of the Lao patients with apparent *R. helvetica* infection had a rash, unlike the 8 patients described previously with *R. helvetica* infection (*5**,**28*). Evidence for acute human infection with *R. felis* has been found in North and South America, Europe, and the Thailand/Burma border (*5**,**31*). The clinical symptoms of the patient described from the Thailand/Burma border were similar to those of our Lao patient, and neither had a rash. Evidence for *R. conorii* has been found in India (*4**,**32*) and on the Thailand/Burma border (*5*). *Rickettsia* "AT1" was originally isolated from Japanese *Amblyomma* ticks, and its genotype is most closely related to rickettsiae from Slovakian *Ixodes* ticks (*33*). The relevance of *Rickettsia* "AT1" to human disease remains uncertain. Although no acute *C. burnetii* infections were found in this series, Q fever has recently been described from northeast Thailand (*34*).

Raised serum creatinine kinase levels have been described in patients with scrub typhus (*35*) and as an apparently nonspecific result of febrile illness (*36*). In a series of patients with fever in Israel, an elevated creatinine kinase level was associated with increased blood urea, low serum phosphate, reduced consciousness, tremor, and muscle tenderness. Alcoholism and high body temperature may also be associated factors (*36*). In Laos, a rise in creatinine kinase level may also have been a consequence of the common practice of administering intramuscular injections before hospital (unpub. data). Because serum creatinine kinase concentrations are higher in patients with rickettsioses who have myalgia than in those without, muscle pain is likely to be associated with mild muscle damage.

This study is of similar design to a recent investigation of the causes of fever in adults living in and around another tropical capital city, Kathmandu, although the Nepalese study included outpatients and sampled 4 months of 1 year (*7*). The frequency of rickettsioses was lower in patients in Kathmandu, with serologic evidence of acute infection with murine typhus in 11% and scrub typhus in 3%. The high incidence of patients in Vientiane who have diseases for which the vectors, such as chiggers and ticks, are likely to be predominantly rural is not surprising. Many inhabitants of the city visit farms in rural areas, and persons with occupations that would not conventionally be regarded as of high risk for rickettsioses may be exposed. In addition, suburban scrub typhus has been described (*37*).

The decision to enter a particular patient into the study was the responsibility of many doctors, and some infected patients may not have been recruited. Only 11% of the Lao population live in the relatively urbanized areas of Vientiane City, and the results of this study are unlikely to be applicable to the rest of the country, which is diverse in geography and ethnicity. A hospital-based study such as this will tend to underestimate the incidence of disease, and infections, such as scrub typhus, which tend to affect farmers, will be more common in rural Laos. Additional limitations of the study are that we did not perform serologic analysis on all patients who did not have a clinically meaningful blood culture during the study period, that the median interval between acute- and convalescent-phase serum samples was relatively short (5 days), and that 49% of patients did not have a convalescent-phase sample.

These data have affected local clinical practice. With the realization that scrub typhus is an important disease, patients' skin surfaces are now routinely completely examined for eschars, and doxycycline therapy is added at an earlier stage for patients with headache, fever, and myalgia. The drugs usually administered for *syndrôme paludéen* were ampicillin or cotrimoxazole, both of which are ineffective against rickettsiae. These results suggest that an antirickettsial agent, such as doxycycline, should be included in the empiric treatment of Lao adults with fevers whose clinical features are consistent with a rickettsiosis. However, analysis of the clinical features of patients in this study with rickettsiosis, leptospirosis, dengue, and typhoid (unpub. data) suggest that these diseases are difficult to distinguish reliably on clinical examination and that rapid, inexpensive diagnostic tests will help guide therapy. An oral drug with high efficacy against uncomplicated rickettsiosis, leptospirosis, and typhoid could be of considerable use. Azithromycin is effective in treating uncomplicated typhoid fever in Vietnam (*38*) and scrub typhus in Korea (*39*), and it may be effective against leptospires in vitro (*40*). In parallel with the adoption of effective artemisinin-based combination therapy for malaria in rural Laos, the need is urgent to develop rapid and inexpensive tests to diagnose alternative causes of fever and to improve the treatment of common nonmalarious fevers.
